# An Explainable Patch-Based Deep Learning Framework for Marker-Specific Computational Assessment of Ovarian Autophagy in Digital Histopathology

**DOI:** 10.3390/bioengineering13101103

**Published:** 2026-09-23

**Authors:** Fatma Nur Kiliçkaya İşci, Celal Öztürk, Eda Köseoğlu, Arzu Hanım Yay

**Affiliations:** 1 Department of Computer Technologies-Computer Programming, Technical Sciences Vocational School, Kayseri University, Kayseri 38280, Türkiye; 2 Department of Computer Engineering, Faculty of Engineering, Erciyes University, Kayseri 38039, Türkiye; 3 Department of Software Engineering, Faculty of Engineering, Erciyes University, Kayseri 38039, Türkiye; 4 Department of Histology and Embryology, Erciyes University, Kayseri 38039, Türkiye

**Keywords:** autophagy, LC3, patch-based learning, deep learning, explainable AI, Grad-CAM, ovarian histopathology, PERGA + PMOS

## Abstract

Autophagy is essential for ovarian physiology and is implicated in pathological conditions such as Polyendocrine Metabolic Ovarian Syndrome (PMOS). The evaluation of autophagy-related markers in histopathological images is often subjective and observer-dependent. This study presents an explainable computational pathology framework that integrates patch-based deep learning with Grad-CAM-based visual interpretation to enable objective and reproducible analysis of autophagy-related immunohistochemical (IHC) staining in rat ovarian tissue. Beclin-1, LC3, and p62-stained rat ovarian tissue images from control, PMOS, bee bread (PERGA), and PERGA + PMOS groups were analyzed using a patch-based deep learning approach. Multiple convolutional neural network architectures, including DenseNet121, EfficientNetV2B0, and ConvNeXtTiny, were trained and evaluated, and their outputs were combined through an ensemble strategy. Image-level predictions were generated by averaging patch-level probabilities, and Grad-CAM was used to visualize image regions influencing model decisions. The LC3-based ensemble model achieved the highest overall image-level classification performance, with an F1-score of 78.28% and an AUC of 88.15%. In contrast, the Beclin-1 DenseNet121 model demonstrated the highest discrimination performance based on AUC, reaching 93.63%. Models trained on p62 images showed lower classification performance, likely due to the heterogeneous staining characteristics of p62 expression. Grad-CAM visualizations revealed that model activation was predominantly localized to DAB-positive and tissue-informative regions, supporting the interpretability of the proposed framework. In summary, this approach enables objective, reproducible, and interpretable analysis of autophagy-related histopathological images. The findings suggest that patch-based deep learning combined with explainable artificial intelligence offers a promising computational tool for assessing autophagy-related alterations in rat ovarian tissue.

## 1. Introduction

Polyendocrine metabolic ovarian syndrome (PMOS), previously referred to as polycystic ovary syndrome (PCOS), is a prevalent endocrine-metabolic disorder that impacts women’s reproductive and metabolic health. PMOS is not an isolated ovarian disease but rather a heterogeneous condition defined by the complex interaction of endocrine, metabolic, and ovarian dysfunctions. These dysfunctions encompass hyperandrogenism, ovulatory dysfunction, impaired follicular maturation, alterations in folliculogenesis, menstrual irregularity, and infertility [1]. Among the cellular mechanisms associated with ovarian dysfunction, autophagy is attracting increasing attention in PMOS research. In addition, Kobayashi et al. emphasized that androgen excess may impair mitochondrial function and disrupt the compensatory regulation of the mitophagy-apoptosis axis in granulosa cells and oocytes, further supporting the relevance of autophagy-related pathways in PMOS pathogenesis [2]. Autophagy is a cellular degradation and recycling process that contributes to cellular homeostasis by removing damaged organelles and proteins. Zhao et al. reported that dysregulated autophagy in PMOS is linked to abnormal ovulation, insulin resistance, hyperandrogenemia, and low-grade inflammation, indicating that autophagy-related mechanisms may contribute to PMOS-associated ovarian dysfunction [3]. In the ovary, autophagy is involved in follicle development, primordial follicle regulation, and follicular atresia. Zhou et al. found that most follicles in the mammalian ovary undergo atresia without reaching ovulation, with both autophagy and apoptosis regulating primordial follicle development and follicular atresia [4]. Consequently, autophagy is a critical cellular mechanism to consider when evaluating PMOS-associated ovarian dysfunction. More recently, Tang et al. emphasized that autophagy has context-dependent roles in ovarian physiology and disorders, contributing to oocyte development, granulosa cell viability, hormone synthesis, ovulation, and luteal function, whereas dysregulated autophagy may be associated with ovarian dysfunctions, including PMOS [5]. Interest in autophagy in the context of PMOS has increased due to its potential roles in follicular cell function, follicle development, anovulation, hyperandrogenemia, and metabolic disorders. Kumariya et al. highlighted that autophagy is involved in the regulation of follicular development during normal ovarian function and that impaired autophagy in follicular cells in PMOS may be associated with disease pathogenesis [6]. Similarly, Cheng et al. suggested that autophagy may be involved in the development and progression of PMOS in various ovarian cell types, including granulosa cells, oocytes, and theca cells [7]. These studies underscore the significance of autophagy as a research focus for understanding ovarian changes associated with PMOS. In a study directly examining autophagic alterations in ovarian tissue related to PMOS, Li et al. reported increased autophagy in both human and rat ovarian tissues, specifically noting elevated LC3B levels and reduced levels of the autophagy substrate SQSTM1/p62 in rat granulosa cells [8]. Another study on granulosa cells demonstrated increases in ATG5, ATG7, and BECN1 mRNA levels and the LC3-II/LC3-I ratio, along with a decrease in SQSTM1/p62 levels. The same study identified a correlation between androgen levels and BECN1 expression, suggesting that androgens may contribute to the activation of autophagy in granulosa cells [9]. Zhang et al. reported that elevated HMGB1 levels are associated with increased autophagy in granulosa cells, as indicated by an increased LC3B-II/I ratio and ATG7 levels, and a decrease in p62 levels [10]. Tong et al. suggested that insulin resistance, granulosa cell autophagy, and apoptosis in the pathogenesis of PMOS may be linked to the PI3K signaling pathway [11]. In a letrozole-induced PMOS rat model, Xu et al. reported that metformin ameliorated ovarian pathological alterations by decreasing excessive autophagy in granulosa cells through the PI3K/AKT/mTOR pathway [12]. Consistent with this pathway-related interpretation, Ma et al. reported that Wnt5a downregulation suppressed autophagy in DHT-induced granulosa cells and a DHEA-induced mouse model through activation of the PI3K/AKT/mTOR pathway, while also improving polycystic ovarian changes [13]. Similarly, Jin et al. reported that berberine combined with metformin inhibited excessive granulosa cell autophagy in a DHEA-induced PMOS rat model through activation of the AMPK/AKT/mTOR pathway, accompanied by decreased Beclin-1 and LC3-II/LC3-I levels and increased p62 expression [14]. Collectively, these findings support the selection of LC3, Beclin-1, and p62 as autophagy-related markers for the immunohistochemical assessment of ovarian tissue alterations associated with PMOS [8,9,10,14]. Zhao et al. identified autophagy as a significant research focus regarding the pathological mechanisms and potential therapeutic targets of PMOS, reporting associations between dysregulated autophagy and abnormal ovulation, insulin resistance, hyperandrogenemia, and low-grade inflammation [3]. These findings support the use of autophagy-related markers, such as LC3, Beclin-1, and p62, as indicators for the immunohistochemical assessment of ovarian changes associated with PMOS. Immunohistochemical evaluation of these markers enables the examination of PMOS-related autophagy patterns at the tissue level. However, traditional immunohistochemistry (IHC) assessment relies primarily on visual interpretation of staining intensity, a process that is time-consuming, semi-quantitative, and susceptible to inter-observer variability. Rizzardi et al. demonstrated that digital image analysis can yield results consistent with visual scoring by pathologists when quantitatively assessing IHC staining intensity [15]. Similarly, Varghese et al. developed a pixel-based IHC analysis approach based on DAB/hematoxylin color separation, reporting that antibody staining intensity can be evaluated in a more objective and quantitative manner [16]. Color variability in histopathological images may arise due to differences in tissue preparation, staining protocols, and scanners. Khan et al. noted that a stain normalization approach based on color deconvolution can mitigate such color variations, thereby contributing to more robust digital histopathology analyses [17]. Quantitative analysis based on the DAB channel and digital image processing methods therefore provides a significant methodological foundation for evaluating IHC images stained for LC3, Beclin-1, and p62. Digital analysis of histopathological images has become a major area of research within computer-aided diagnostic approaches in recent years. Banerji et al. noted that while convolutional neural networks (CNNs) are widely used for classifying histopathological images, the field faces challenges such as color variability, extremely high resolution, and insufficient labeling [18]. Recent reviews have further emphasized that computational pathology still faces key challenges related to gigapixel image processing, limited labeled data, domain shifts across institutions, and the interpretability of machine learning models [19]. These challenges suggest that evaluating selected tissue-informative regions is more appropriate than analyzing entire images directly. Consistent with this perspective, Gadermayr and Tschuchnig emphasized that digital pathology images often contain extensive image information while detailed patch-, region-, or pixel-level annotations are limited, supporting the use of region-based learning strategies in histopathological image analysis [20]. Patch-based approaches enable the examination of local patterns within histopathological images, reduce computational load, and make the learning process more manageable. The interpretability of model decisions is as important as classification performance in medical image analysis. In immunohistochemical images, the ability to visualize which tissue regions the model focuses on can help establish a clearer relationship between computational analysis and histopathological patterns. This study proposes a computational framework that integrates DAB-channel-based quantitative analysis, patch-based deep learning, and Grad-CAM-based visual explainability approaches to evaluate images of rat ovarian tissue stained for LC3, Beclin-1, and p62.

In the context of the experimentally induced PMOS model and bee bread (PERGA) administration, there is a need for more objective, quantitative, and interpretable analysis approaches to evaluate autophagy-related changes in ovarian tissue [21]. Although immunohistochemical evaluation of autophagy-related markers such as LC3, Beclin-1, and p62 is widely used, these assessments are subject to observer-dependent interpretation and may not adequately capture the heterogeneity of local staining within tissue regions. To address these limitations, this study introduces an integrated computational analysis framework for evaluating autophagy-related histopathological patterns in rat ovarian tissue. The framework incorporates DAB-channel-based quantitative staining analysis, patch-based deep learning, and Grad-CAM-based visual explainability. Quantitative staining features were extracted from IHC images stained for LC3, Beclin-1, and p62; local histopathological patterns were identified from selected tissue patches; and image regions influencing model predictions were visualized using Grad-CAM. This computational approach enables a more objective, quantitative, and interpretable evaluation of autophagy markers in the context of PMOS and PERGA applications.

## 2. Materials and Methods

This section describes the proposed autophagy image analysis framework, from dataset development to model interpretability. First, the dataset, experimental groups, and immunohistochemical images of the autophagy markers Beclin-1, LC3, and p62 are defined. Then, image preprocessing, patch-based image representation, and quantitative DAB analysis steps are presented. Following this, the deep learning models used, the training strategy, the image-level prediction approach, and the evaluation metrics are explained. Finally, a Grad-CAM-based explainability analysis is performed to biologically interpret the textural regions the model focuses on in its decision-making.

### 2.1. Dataset

In this study, female rats were used, and the animals were randomly divided into 4 groups: control, PMOS, PERGA, and PERGA + PMOS. The control group was untreated, while the other groups underwent the relevant experimental procedures. All animals were housed under the same environmental and care conditions throughout the study. Immunohistochemical ovarian images of autophagy-related markers Beclin-1, LC3, and p62 were used in the study. Histopathological sections were imaged using an Olympus BX51 light microscope (Olympus Corp., Tokyo, Japan). All images were obtained at a magnification of 20 µm and have a spatial resolution of approximately 0.100 µm/pixel. A total of 659 immunohistochemical images were obtained. The obtained images underwent quality control; unreadable, distorted, or images without analyzable tissue were removed from the dataset. After cleaning, the dataset was organized into three different autophagy markers (Beclin-1, LC3, and p62) and four experimental groups (control, PMOS, PERGA, and PERGA + PMOS). To create a standard input size suitable for deep learning models, all images were processed using a patch-based approach. In this context, patches containing 224 pixels of texture were extracted from histopathological images. Empty background regions and areas with low information were automatically eliminated, and only patches with significant texture were included in the analysis. As a result of this process, a total of 7908 patches were obtained. The dataset was divided into training (70%), validation (15%), and testing (15%) subsets using a stratified approach that preserved the class distribution. Marker-based image distributions, data splitting statistics, and patch generation information are presented in Table 1.

To reduce the risk of patch-level data leakage, dataset partitioning was conducted at the level of original histological images before patch-based model training and evaluation. For each autophagy marker, clean immunohistochemistry (IHC) images were allocated to training, validation, and testing subsets using a stratified splitting strategy based on experimental group labels, as summarized in Table 1. After this image-level partitioning, all patches derived from a given histological image were assigned to the same subset as their parent image. As a result, patches from the same original histological image were retained exclusively within a single subset and were not distributed across the training, validation, or testing sets. This approach ensured that patch-level learning and image-level performance evaluation were performed on mutually independent original histological images, thereby enabling a more robust interpretation of the reported classification performance.

### 2.2. Autophagy Markers

Autophagy is a process of degradation and recycling of damaged organelles, misfolded proteins, and metabolic substrates by lysosomal degradation [21]. It is therefore defined as an evolutionarily conserved catabolic process involved in the regulation of cellular homeostasis, energy balance, and stress responses [22]. This process plays a critical role in ovarian tissue, regulating cellular homeostasis, energy balance, the oxidative stress response, and follicular development. The literature reports that dysregulation of autophagic mechanisms is associated with PMOS, granulosa cell damage, and follicular dysfunction [6,21,23]. Therefore, evaluating autophagy-related biomarkers provides important biological insights into ovarian changes associated with PMOS.

This study aimed to comprehensively analyze the initiation, autophagosome formation, and degradation stages of the autophagic process by evaluating the markers Beclin-1, LC3, and p62 simultaneously (Figure 1). Furthermore, evaluating different staining patterns of these markers using patch-based deep learning models and explainable artificial intelligence methods enabled a biologically interpretable examination of autophagy-related changes in ovarian tissue.

#### 2.2.1. Beclin-1

Beclin-1 is a key regulatory protein involved in the initiation phase of autophagy and plays a role in the formation of autophagosomes. Therefore, changes in Beclin-1 expression are associated with early autophagic activation [23].

#### 2.2.2. LC3

LC3 is one of the most common autophagy markers associated with the autophagosome membrane and is considered a gold standard marker for evaluating autophagic activity. The conversion of LC3-I to the membrane-bound LC3-II form during autophagy results in distinct punctate staining patterns in immunohistochemical images. This distinct spatial localization creates distinctive morphological patterns for patch-based deep learning models.

#### 2.2.3. p62

p62 is one of the adaptor proteins that transport ubiquitinated proteins to autophagosomes and is degraded during autophagic degradation. Therefore, p62 accumulation is often associated with impaired autophagic flow. The more heterogeneous distribution of p62 staining creates more complex patterns for image-based classification.

### 2.3. Patch-Based Image Processing

Histopathological images were processed using a patch-based approach to enable deep learning models to learn local morphological patterns more effectively. In this context, high-resolution immunohistochemical ovarian images were divided into sub-images (patches) with a pixel size of 224×224. Two key criteria were used in the patch selection process to ensure the inclusion of biologically significant regions in the analysis: tissue ratio and DAB staining intensity. The tissue ratio is the ratio of the actual histological tissue area within the patch to the patch’s total area. DAB staining intensity indicates the degree of immunohistochemical positivity for autophagy markers. Based on these criteria, patches with large empty background areas, low tissue density, or insufficient DAB staining were excluded from the analysis. Thus, only patches containing biologically informative and autophagy-related morphological patterns were incorporated into the model.

Patch selection was performed using a reproducible quality-based filtering procedure that combined tissue content and DAB staining information. Candidate patches were extracted from each clean IHC image using a sliding-window approach with a patch size of 224 × 224 pixels and a stride of 112 pixels. For each candidate patch, tissue content was estimated by converting the RGB patch to grayscale and excluding near-background regions. Pixels with grayscale intensity values lower than 225 were considered tissue pixels, and the tissue ratio was calculated as the proportion of tissue pixels within the patch.

DAB staining information was obtained using H-DAB color deconvolution. The DAB channel was evaluated only within the tissue mask. To define DAB-positive pixels, a patch-specific threshold was calculated as the 75th percentile of DAB-channel values among tissue pixels. Pixels exceeding this threshold within the tissue mask were considered DAB-positive. The DAB-positive ratio was then calculated as the proportion of DAB-positive pixels within the patch, and the mean DAB intensity was computed from the DAB-positive pixels.

Candidate patches were retained if they had a tissue ratio of at least 0.35 and a DAB-positive ratio of at least 0.03. Each retained patch was ranked using the following quality score:

Candidate patches were ranked according to the quality score defined in Equation (1):

(1) $$Q_{\mathrm{patch}}=R_{\mathrm{tissue}}+4R_{\mathrm{DAB}}+I_{\mathrm{DAB}},$$

where $Q_{\mathrm{patch}}$ denotes the patch quality score, $R_{\mathrm{tissue}}$ represents the tissue ratio, $R_{\mathrm{DAB}}$ denotes the DAB-positive ratio, and $I_{\mathrm{DAB}}$ represents the mean DAB intensity.

The thresholding criteria and scoring formulation used for patch selection are summarized in Table 2.

For each original histological image, up to 12 patches with the highest quality scores were selected for subsequent patch-based classification. If no candidate patch satisfied the DAB-positive criterion, the selection was performed among tissue-rich patches that met the minimum tissue-ratio threshold. This approach reduces computational costs and enables the model to focus on the tissue’s distinctive morphological features. The general flow of the patch extraction and selection process is shown in Figure 2.

### 2.4. Quantitative DAB Analysis

DAB-based images obtained after immunohistochemical (IHC) staining were used to evaluate the expression intensity and distribution of autophagy-related markers (Beclin-1, LC3, and p62) in ovarian tissue. It is assumed that as the brown intensity of DAB staining increases, the expression level of the relevant protein in the tissue also increases. In this study, these staining patterns were quantitatively analyzed using image processing-based methods. In this context, two basic measurement parameters were calculated:

- Marker-positive object count: This represents the total number of associated regions or objects showing DAB-positive staining. This metric shows how many distinct regions in the tissue the marker is expressed in.
- DAB-positive area (%): This represents the ratio of DAB-positive areas to the total tissue area. This measurement shows the prevalence and the area of positive staining in the tissue.

Statistical analysis was performed to evaluate group-wise differences in quantitative DAB measurements for each autophagy marker. The analyzed DAB-related parameters included DAB-positive area, mean DAB intensity within tissue regions, mean DAB intensity within DAB-positive regions, and integrated optical density. Since these image-derived measurements may not necessarily follow a normal distribution, the Kruskal-Wallis test was used as the primary non-parametric test to compare experimental groups. One-way ANOVA was also performed as a complementary parametric analysis. Statistical significance was defined as p<0.05. The corresponding *p*-values were reported to support the interpretation of significant differences among experimental groups.

Sample histopathological images and marker-based quantitative analysis results for the dataset are presented in Figure 3. The marker-positive object count parameter represents the numerical distribution of positive staining areas, while the DAB-positive area (%) value quantitatively reveals the level of spread of the relevant marker within the tissue.

Figure 3 shows the differences in immunohistochemical staining between the experimental groups; furthermore, the percentages of DAB-positive areas and the densities of marker-positive regions are compared quantitatively. Particularly in LC3-stained images, distinct punctate staining patterns were observed, while more heterogeneous and irregular distributions were seen in p62 images. These visual differences were evaluated for their direct contribution to the classification performance of deep learning models. The obtained density information was used in patch selection and quality control processes.

### 2.5. Deep Learning Models

Three pretrained convolutional neural network architectures—DenseNet121, EfficientNetV2B0, and ConvNeXtTiny—were employed for patch-based image classification. Model training and evaluation were implemented using TensorFlow/Keras within custom Python scripts. These models were selected to facilitate a comparison of distinct convolutional feature-extraction strategies. DenseNet121 utilizes dense connectivity, enabling feature maps from earlier layers to be reused by subsequent layers, which supports both feature propagation and reuse [24]. EfficientNetV2B0 is a compact architecture that balances model depth, width, and input resolution to enhance computational efficiency [25]. ConvNeXtTiny is a contemporary convolutional architecture that integrates design principles from recent deep learning models while maintaining a convolution-based framework [26]. Collectively, these architectures offer complementary backbone designs for the evaluation of marker-specific histopathological image classification. The overall model structure is presented in Figure 4. All models were initialized using ImageNet pretrained weights. The original classification heads were replaced with a sequence of global average pooling, batch normalization, dropout, a fully connected layer, and a final softmax classification layer. The classification head specifically comprised global average pooling, batch normalization, dropout, a dense layer with 256 units and ReLU activation, a second batch normalization layer, dropout, and a final dense layer with softmax activation for four-class classification.

Model training was conducted in two stages. Initially, the pretrained backbone was frozen while only the newly added classification layers were trained. Subsequently, fine-tuning was performed by unfreezing the final layers of the backbone. Data augmentation techniques, including random horizontal and vertical flipping, brightness adjustment, contrast adjustment, and rotation, were applied during training. Class imbalance was mitigated using class weights. The Adam optimizer and categorical cross-entropy loss function were employed for model optimization.

The image-level ensemble prediction procedure is outlined in Algorithm 1. For each marker, convolutional neural network (CNN) models generated class-probability vectors for the extracted patches. For each original histological image, patch-level probability vectors corresponding to the same image were averaged to produce an image-level probability vector for each model. The ensemble prediction was generated by averaging the image-level probability vectors from DenseNet121, EfficientNetV2B0, and ConvNeXtTiny. The final predicted class was determined by selecting the class with the highest averaged probability value. Only probability vectors were used in the ensemble process; hard class labels were not averaged.
**Algorithm 1:** Image-level Ensemble Prediction Procedure  
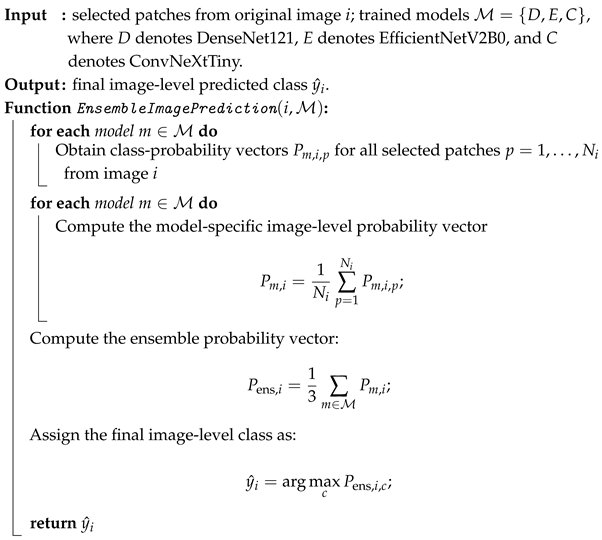


#### 2.5.1. Performance Evaluation Metrics

Model performance was evaluated using accuracy, precision, recall, F1-score, and the area under the receiver operating characteristic (ROC) curve (AUC). These metrics were used to measure different aspects of classification performance. True positive (TP), true negative (TN), false positive (FP), and false negative (FN) values were considered in the calculations.

The accuracy metric is given in Equation (2). It represents the ratio of correctly classified samples to the total number of samples.

(2) $$\mathrm{Accuracy}=\frac{\mathrm{TP}+\mathrm{TN}}{\mathrm{TP}+\mathrm{TN}+\mathrm{FP}+\mathrm{FN}}$$

The precision metric is given in Equation (3). It shows how many of the model’s predicted positives are actually positive.

(3) $$\mathrm{Precision}=\frac{\mathrm{TP}}{\mathrm{TP}+\mathrm{FP}}$$

The recall metric is given in Equation (4). It shows how many of the true positive samples the model correctly identified.

(4) $$\mathrm{Recall}=\frac{\mathrm{TP}}{\mathrm{TP}+\mathrm{FN}}$$

The F1-score is given in Equation (5). It represents the harmonic mean of precision and recall and provides a more reliable performance assessment, especially on unbalanced datasets.

(5) $$F_{1}=2\times \frac{\mathrm{Precision}\times \mathrm{Recall}}{\mathrm{Precision}+\mathrm{Recall}}$$

The AUC metric represents the area under the receiver operating characteristic (ROC) curve. This metric was used to evaluate the model’s discrimination performance between classes. An AUC value closer to 1 indicates better classification performance.

#### 2.5.2. Grad-CAM-Based Explainability Analysis

Gradient-weighted Class Activation Mapping (Grad-CAM) was used as a post-hoc visual explainability method to examine the image regions contributing to model predictions. In this study, Grad-CAM analysis was applied to representative test patches selected for each marker and experimental group. The heatmaps were generated using the last convolutional layer of the trained model and the predicted class output.

For a given class *c*, let $A^{k}$ denote the *k*th feature map in the last convolutional layer and $y^{c}$ denote the corresponding class score (Equation (6)). The importance coefficient of each feature map for class *c* was calculated as follows:

(6) $$\alpha_{k}^{c}=\frac{1}{Z}\sum_{i}\sum_{j}\frac{\partial y^{c}}{\partial A_{ij}^{k}},$$

where $A_{ij}^{k}$ represents the activation value of the *k*th feature map at spatial location (i,j), and *Z* denotes the total number of spatial locations in the feature map (Equation (7)). The class-specific Grad-CAM localization map was then obtained as:

(7) $$L_{\mathrm{Grad}-\mathrm{CAM}}^{c}=\mathrm{ReLU}\left(\sum_{k}\alpha_{k}^{c}A^{k}\right).$$

The ReLU operation retains the regions that positively contribute to the predicted class. The resulting heatmap was resized to the input patch size and overlaid on the original patch image for visualization (Figure 5). In this study, Grad-CAM was used to assess whether model attention was localized to tissue-informative and DAB-positive regions rather than background areas or staining artifacts.

## 3. Results

This section presents the quantitative analysis, classification, and annotated AI performance results of the proposed patch-based deep learning framework. First, the results of a DAB-based quantitative analysis of immunohistochemical images of the Beclin-1, LC3, and p62 autophagy markers are evaluated. Then, the classification performances of DenseNet121, EfficientNetV2B0, and ConvNeXtTiny models are compared using accuracy, precision, recall, F1-score, and AUC metrics. Finally, biologically significant regions that the models focus on in their decision-making processes are visualized and interpreted using Grad-CAM-based annotated AI analyses.

### 3.1. Quantitative DAB-Based Marker Analysis

Immunohistochemical DAB staining revealed that the expression patterns of Beclin-1, LC3, and p62 differed among the experimental groups. Marker expression was evaluated using an image processing-based automated quantitative analysis approach, including marker-positive associated region count, DAB-positive area, mean DAB intensity within tissue regions, mean DAB intensity within DAB-positive regions, and integrated optical density. Group-wise statistical comparisons were performed for each marker and quantitative DAB parameter. Statistical significance was defined as p<0.05. Quantitative DAB analysis demonstrated statistically significant differences among the experimental groups for all evaluated autophagy markers and DAB-related measurements, as summarized in Table 3.

The quantitative DAB staining parameters are presented in Figure 6. These measurements include tissue-normalized DAB intensity, mean DAB intensity within DAB-positive regions, and integrated optical density for Beclin-1, LC3, and p62 across the experimental groups. The obtained values were automatically calculated using the image processing-based quantitative analysis pipeline and should not be interpreted as direct manual cell counts. These findings indicate that the quantitative DAB parameters captured marker-specific staining differences across the experimental groups. Overall, changes in the expression patterns of autophagy-related markers were observed in the PMOS group, whereas PERGA-treated groups showed relatively more balanced staining profiles. In particular, some DAB-related parameters in the PERGA + PMOS group tended to approach the control group pattern, suggesting a potential regulatory effect of PERGA treatment on autophagy-related ovarian tissue alterations.

### 3.2. Classification Performance

Four-class classification of Beclin-1, LC3, and p62 markers was performed using patch-based image representation. Model performance was evaluated using accuracy, precision, recall, F1-score, and ROC-AUC metrics. Final image-level predictions were calculated by combining the patch probabilities from the same image.

The results obtained in Table 4 show differences in classification performance among the markers. In particular, LC3 images provided higher performance in differentiating experimental groups.

All models were compared in terms of accuracy, precision, recall, F1-score, and AUC metrics. Among all marker-model combinations, the highest classification performance was achieved by the LC3-based ensemble model, which achieved an F1 score of 78.28% and an AUC of 88.15%. While similar results were obtained with the ensemble model for Beclin-1, the DenseNet121 architecture achieved the highest AUC of 93.63%. In contrast, models trained on p62 performed significantly worse across all metrics. Overall, LC3 consistently outperformed Beclin-1 and p62 across different architectures, demonstrating that LC3 provides more distinctive imaging features in classifying ovarian tissue conditions. Despite the high AUC values, the fact that they are lower than the F1 scores indicates that the model has learned to distinguish between classes but struggles with precise class assignment, especially within morphologically similar groups.

#### 3.2.1. ROC-AUC Analysis

ROC-AUC analysis was used to evaluate the capacity of the models to differentiate between the four experimental groups. The ROC-AUC analysis is given in Figure 7.

In the ensemble model of the LC3 marker, ROC curves showed a significant capacity for discrimination between classes. This indicates that LC3-based image features carry strong information not only for class prediction but also for probabilistic discrimination between groups.

#### 3.2.2. Confusion Matrix Analysis

The confusion matrix of the LC3-based ensemble model shows that the classification success is not homogeneous among the groups in Figure 8.

It was observed that the control group was more clearly separated than the other experimental groups. Mixing occurred between the PMOS and PERGA + PMOS groups. This suggests that histological changes originating from PMOS may contain common morphological features that do not completely separate after PERGA application. Interclass confusion was observed, particularly between biologically close groups.

#### 3.2.3. Model and Marker Comparison

Model comparison results are given in Figure 9. The ensemble approach has been shown to offer more stable performance compared to individual deep learning models. When evaluated by F1 score, results with the LC3 marker were higher than those with Beclin-1 and p62. In the study conducted by [21], LC3 expression levels were reported to be lower than those of Beclin-1. However, the findings of the present study indicate that LC3 staining patterns exhibit more distinct and discriminative image features that support the differentiation among experimental groups. This suggests that, despite its lower expression level, LC3 may serve as a valuable histopathological marker for distinguishing group-specific alterations in digital image-based analysis.

In addition, p62 is a selective autophagy adaptor that is degraded during the autophagic process; therefore, its staining pattern may display a relatively heterogeneous distribution depending on autophagic flux and degradation activity. Accordingly, lower p62-related image features were expected in the present analysis. Consistent with this assumption, our findings demonstrated that p62 exhibited lower values compared with the other autophagy markers, supporting the interpretation that p62 degradation-related heterogeneity may reduce its discriminative image intensity across experimental groups.

### 3.3. Explainability Analysis with Grad-CAM

Grad-CAM visualizations were generated to examine the image regions contributing to the model predictions, as shown in Figure 10. For each experimental group, representative LC3 patches were selected from the test set according to the highest patch quality score. The Grad-CAM heatmap was computed using the last convolutional layer of the trained model and the predicted class output. The resulting heatmap was resized to the input patch size and overlaid on the original patch image to visualize model-attended regions.

Overall, the Grad-CAM analysis supported the interpretability of the proposed framework by visualizing the image regions used by the model during prediction. Since the representative patches were selected using the patch quality score, the visual explanations were generated from tissue-rich and staining-informative image regions. Therefore, Grad-CAM was used as an explainability tool to assess model attention within selected histopathological patches, while biological interpretation was considered together with the staining context and quantitative DAB analysis.

## 4. Discussion

This study assessed the analyzability of autophagy-related ovarian immunohistochemical images using a patch-based deep learning approach combined with explainable artificial intelligence techniques. The findings have significant implications for both technical methodology and histopathological interpretation. Comparative analysis demonstrated that interpretation of model performance varied depending on the evaluation metric employed. The LC3-based ensemble model achieved the highest image-level classification performance by F1-score (78.28%) and an AUC of 88.15%. In contrast, the Beclin-1 DenseNet121 model attained the highest AUC (93.63%) but a lower F1-score (73.44%). This distinction indicates that the LC3-based ensemble provided the most balanced final class-label prediction, whereas Beclin-1 DenseNet121 exhibited stronger threshold-independent discrimination as measured by AUC. The divergence between AUC and F1-score was also evident in class-wise performance. For Beclin-1 DenseNet121, the control and PMOS classes demonstrated relatively stronger classification, while the PERGA and PERGA + PMOS classes had lower recall and F1-scores. These results suggest that Beclin-1-derived features supported general class discrimination, but certain treatment-related groups were more challenging to classify accurately in the four-class setting. In contrast, the LC3-based ensemble displayed a more balanced class-wise profile, with strong performance for the control and PERGA groups and moderate performance for the PMOS and PERGA + PMOS groups. This pattern accounts for the higher F1-score of the LC3 ensemble despite its lower AUC compared to Beclin-1 DenseNet121. The reduced performance of p62-based models was further emphasized in class-level evaluations. While the p62 ensemble model classified the control group effectively, its performance declined substantially for the PMOS, PERGA, and PERGA + PMOS groups. This finding suggests that the limited overall performance of p62 was primarily attributable to decreased separability among non-control experimental groups. This trend may be explained by the heterogeneous staining characteristics of p62 and the complex biological role of p62/SQSTM1 in autophagy, as p62 accumulation may reflect either altered autophagic degradation or impaired autophagic flux rather than a single, uniform change in autophagy activity. LC3 is directly involved in autophagosome formation, which can result in more localized and visually distinctive staining patterns in immunohistochemical images. These findings should be interpreted as evidence of classification-relevant image features rather than as direct confirmation of increased autophagic activity. LC3 should therefore be considered the most discriminative marker for image-based classification within the evaluated dataset, but not as a comprehensive marker for direct assessment of autophagy activity. While the LC3-based ensemble achieved the highest overall image-level F1-score, the confusion matrix revealed class-specific overlap between the PMOS and PERGA + PMOS groups. This outcome indicates that the framework effectively captured LC3-related image features, yet distinguishing treatment-associated alterations from PMOS-related staining patterns remained challenging. The lower performance observed for p62 should also be interpreted with caution, as p62 staining may reflect complex and heterogeneous autophagy-related processes across experimental groups. The patch-based learning approach was central to the proposed framework. By selecting tissue-rich regions with DAB staining signals instead of analyzing entire histopathological images, the model focused on informative tissue areas and minimized the influence of background, empty spaces, and staining artifacts. Aggregating patch-level predictions into image-level predictions further improved the stability of classification outputs. Grad-CAM analyses enhanced the visual interpretability of model decisions. The activation maps were predominantly localized to DAB-positive and tissue-informative regions, demonstrating that the models utilized relevant histopathological image areas during classification. However, these activation patterns should be regarded as evidence of model attention rather than as direct confirmation of biological significance. The PERGA-related findings also require cautious interpretation. The observed image-derived differences may indicate treatment-associated alterations in autophagy marker staining patterns, but they should not be considered direct mechanistic evidence of a protective biological effect. The proposed approach should thus be regarded as an objective computational support tool for evaluating autophagy-related histopathological patterns, rather than as a definitive method for resolving treatment-specific autophagy alterations. This limitation underscores the need for larger datasets, external validation, and complementary quantitative or molecular analyses in future studies.

## 5. Conclusions

This study introduces a computational pathology approach that integrates DAB-channel-based quantitative analysis, patch-based deep learning, and explainable artificial intelligence for analyzing autophagy-related immunohistochemical images in rat ovarian tissue. Model performance varied according to the specific autophagy marker and evaluation metric. The LC3-based ensemble model achieved the most balanced image-level classification performance based on F1-score, while the Beclin-1 DenseNet121 model demonstrated the highest discrimination according to the area under the curve (AUC). In contrast, models based on p62 exhibited lower image-level separability. The patch-based learning strategy allowed the model to focus on tissue-informative regions, and Grad-CAM analyses enhanced the visual interpretability of model decisions by highlighting image regions relevant to classification.The findings of this study suggest that PERGA may exert regulatory and potentially protective effects against PMOS associated autophagic dysregulation. Moreover, the results revealed that the proposed analytical method based on image processing could objectively and reproducibly assess autophagy-related histopathological changes. Future studies with larger datasets and segmentation-based analyses are warranted to improve model performance and enhance clinical applicability.

## Figures and Tables

**Figure 1:**
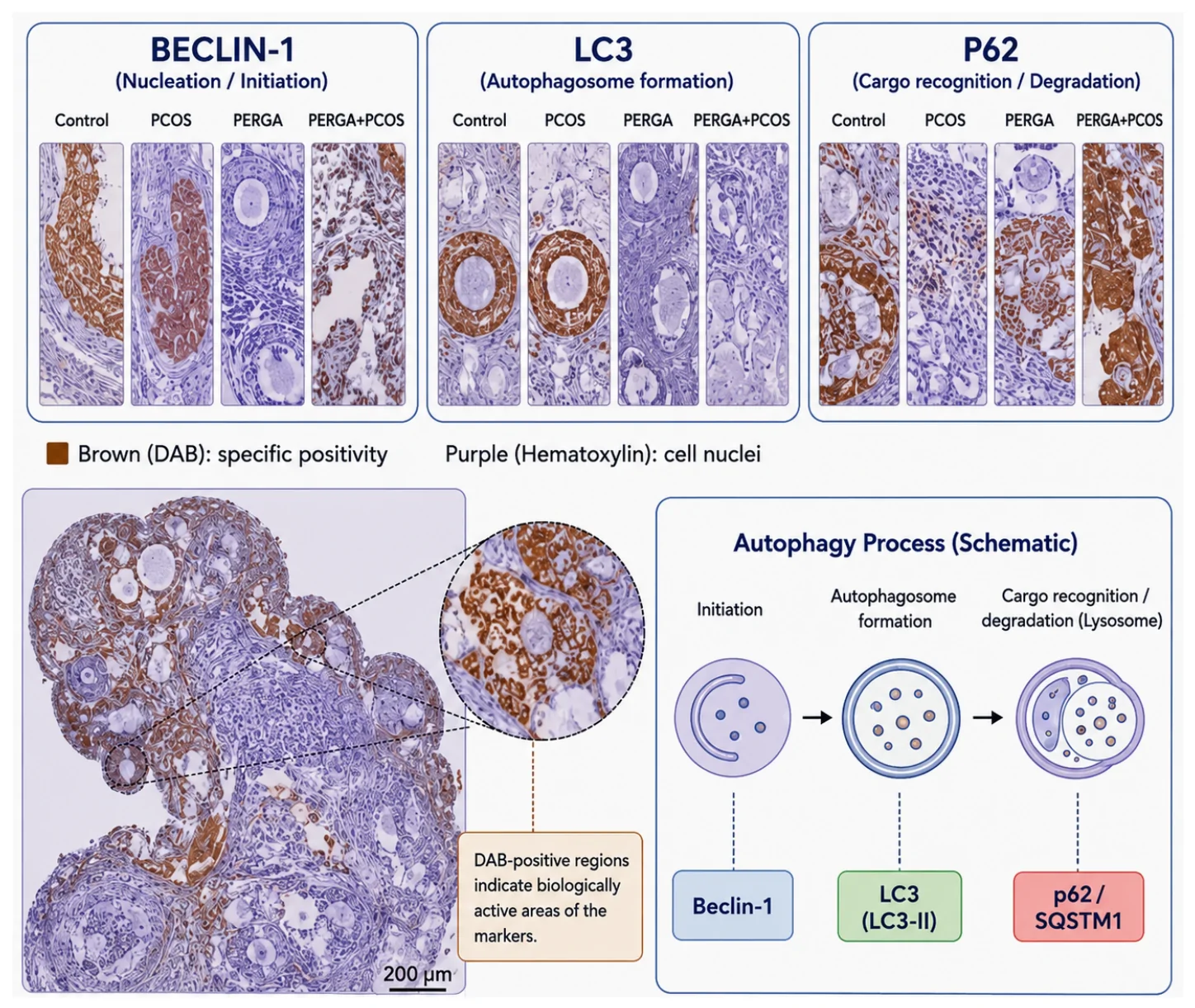
Biological basis of autophagy markers.

**Figure 2:**
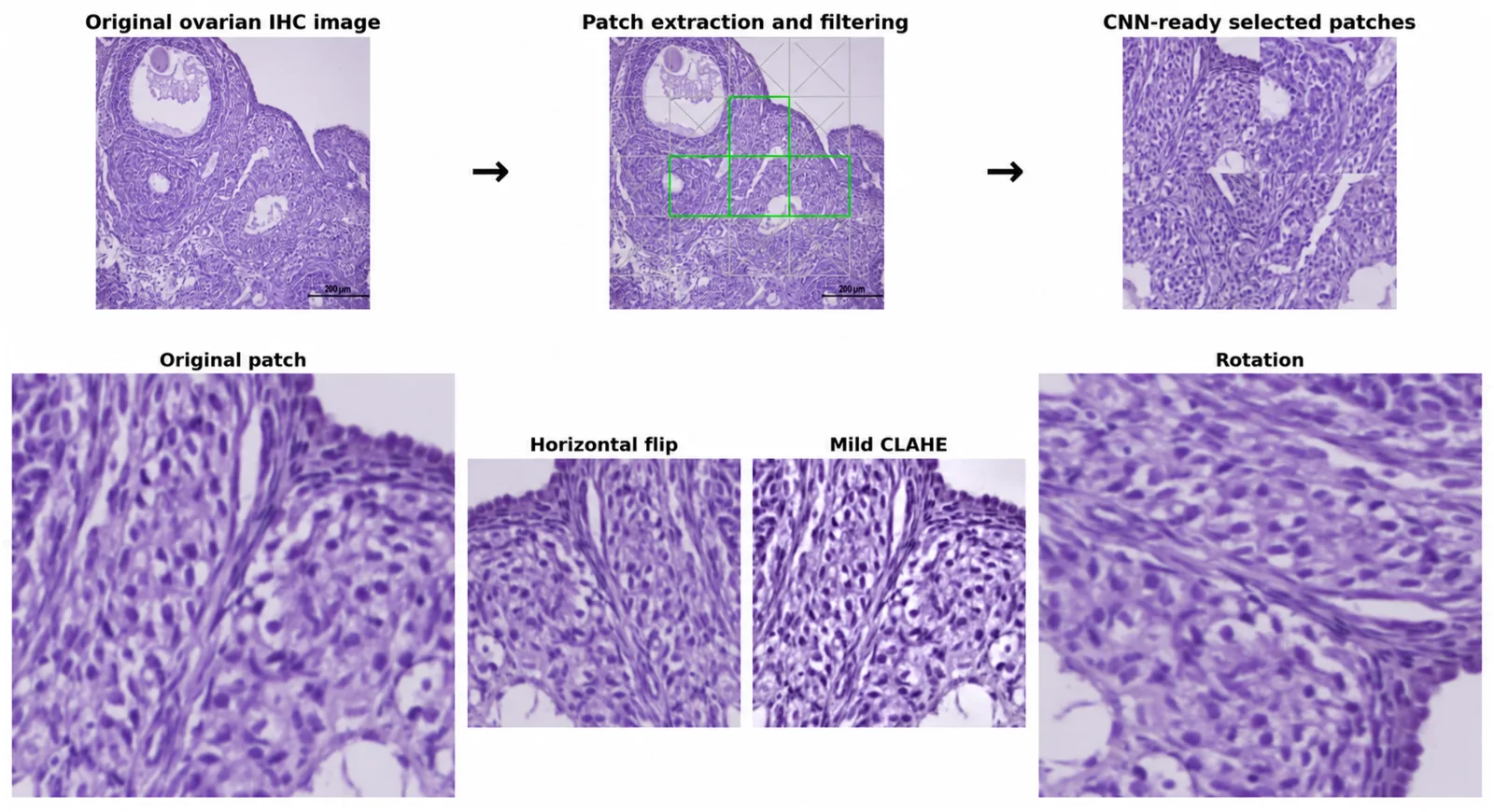
Overview of the proposed patch-based preprocessing workflow. Whole ovarian immunohistochemical images were divided into fixed-size patches of 224 × 224 pixels. In the patch extraction panel, green outlines denote patches retained after tissue- and staining-aware quality filtering, whereas gray outlines represent candidate patch locations that were not selected for subsequent CNN analysis. Selected informative patches were then used for augmentation and CNN-based feature learning.

**Figure 3:**
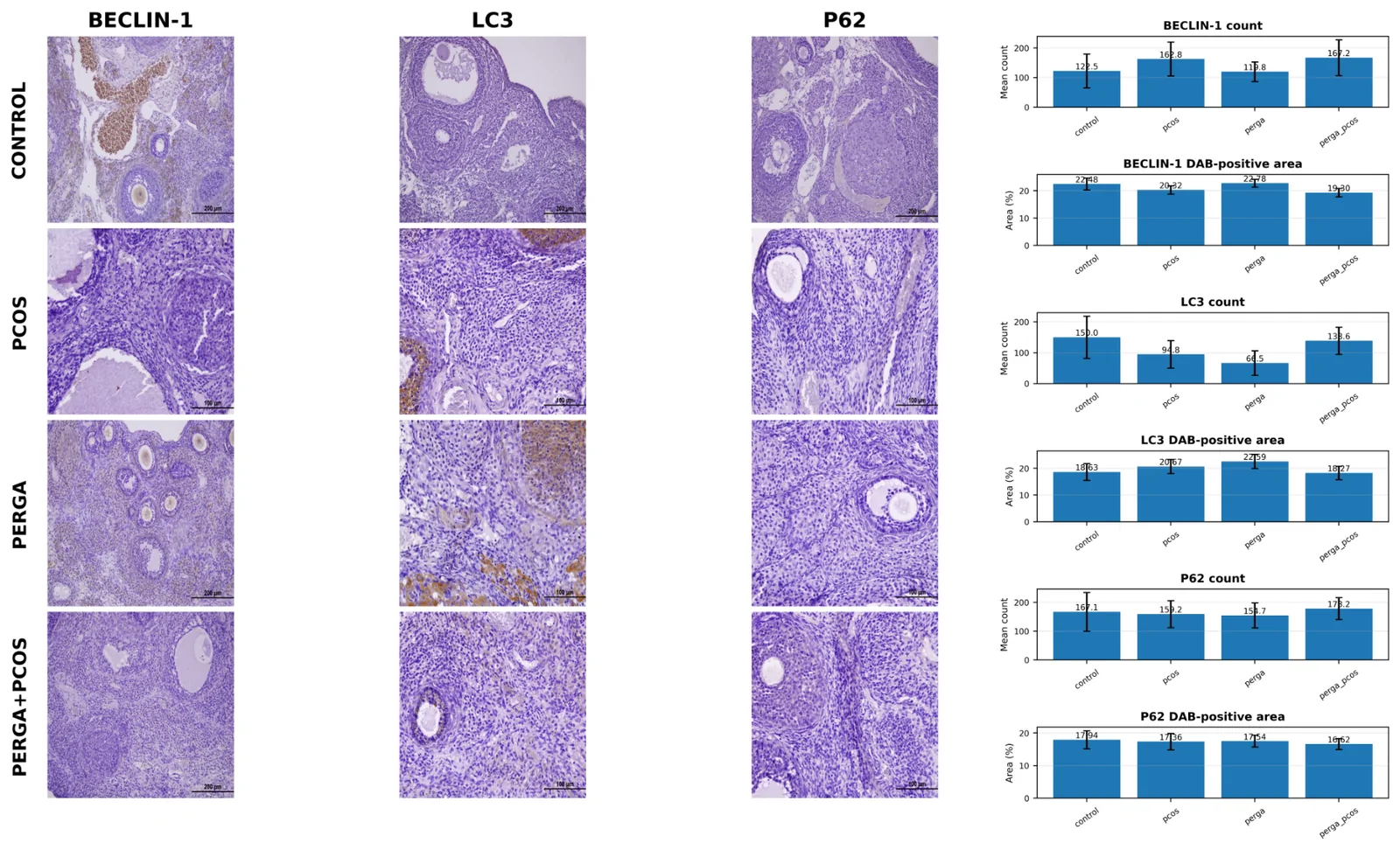
Representative ovarian immunohistochemical images for Beclin-1, LC3, and p62 across experimental groups, together with corresponding DAB-based quantitative summaries. Brown staining indicates DAB-positive immunoreactivity, whereas purple staining represents hematoxylin counterstaining. Blue bars show group-wise quantitative values, and black error bars indicate variability.

**Figure 4:**
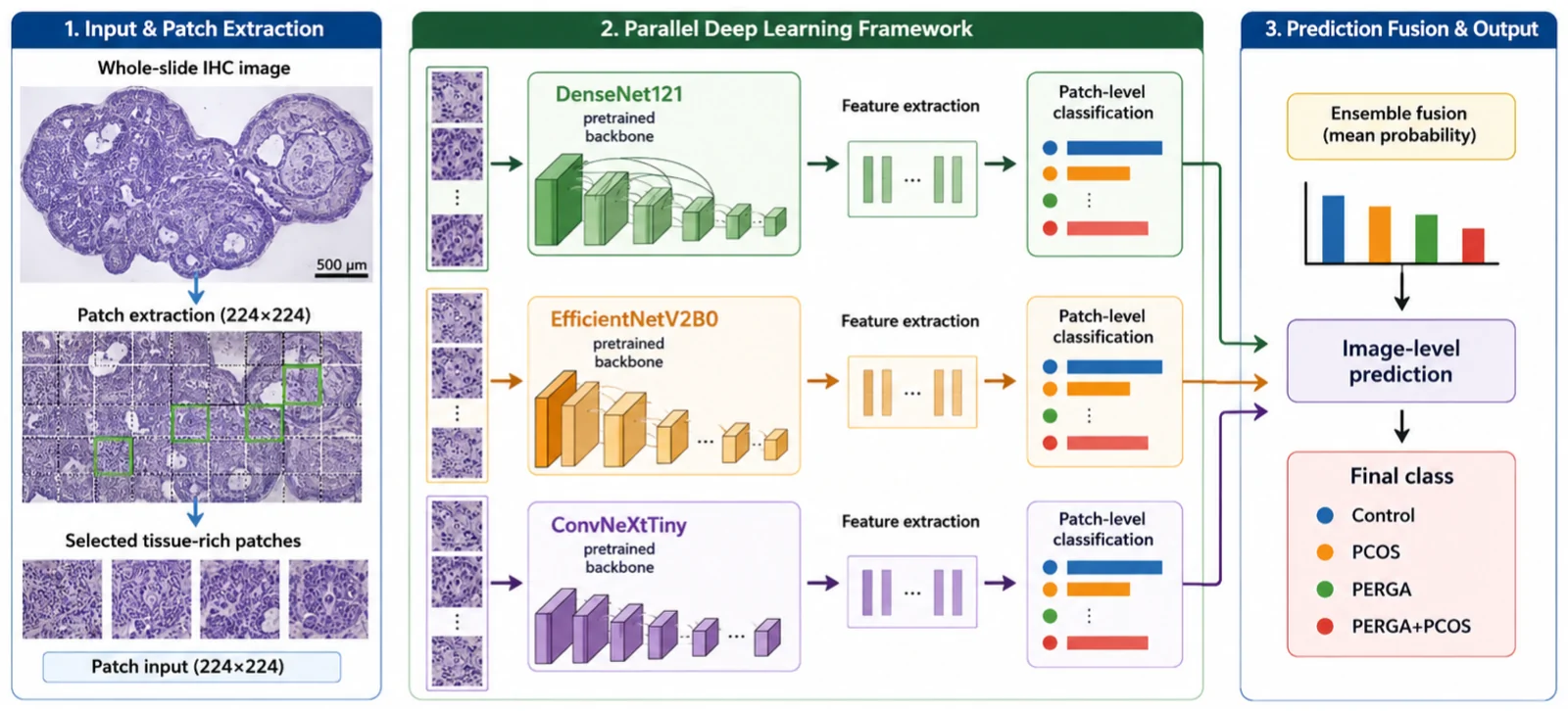
Patch-based parallel deep learning architecture for ovarian IHC image classification. Whole-slide IHC images were divided into 224 × 224-pixel patches, and selected tissue-rich patches were processed in parallel using DenseNet121, EfficientNetV2B0, and ConvNeXtTiny backbones. Green, orange, and purple arrows indicate the DenseNet121, EfficientNetV2B0, and ConvNeXtTiny branches, respectively. Patch-level predictions were fused by mean-probability ensemble aggregation to obtain the final image-level prediction. Ellipses indicate omitted intermediate elements for schematic compactness.

**Figure 5:**
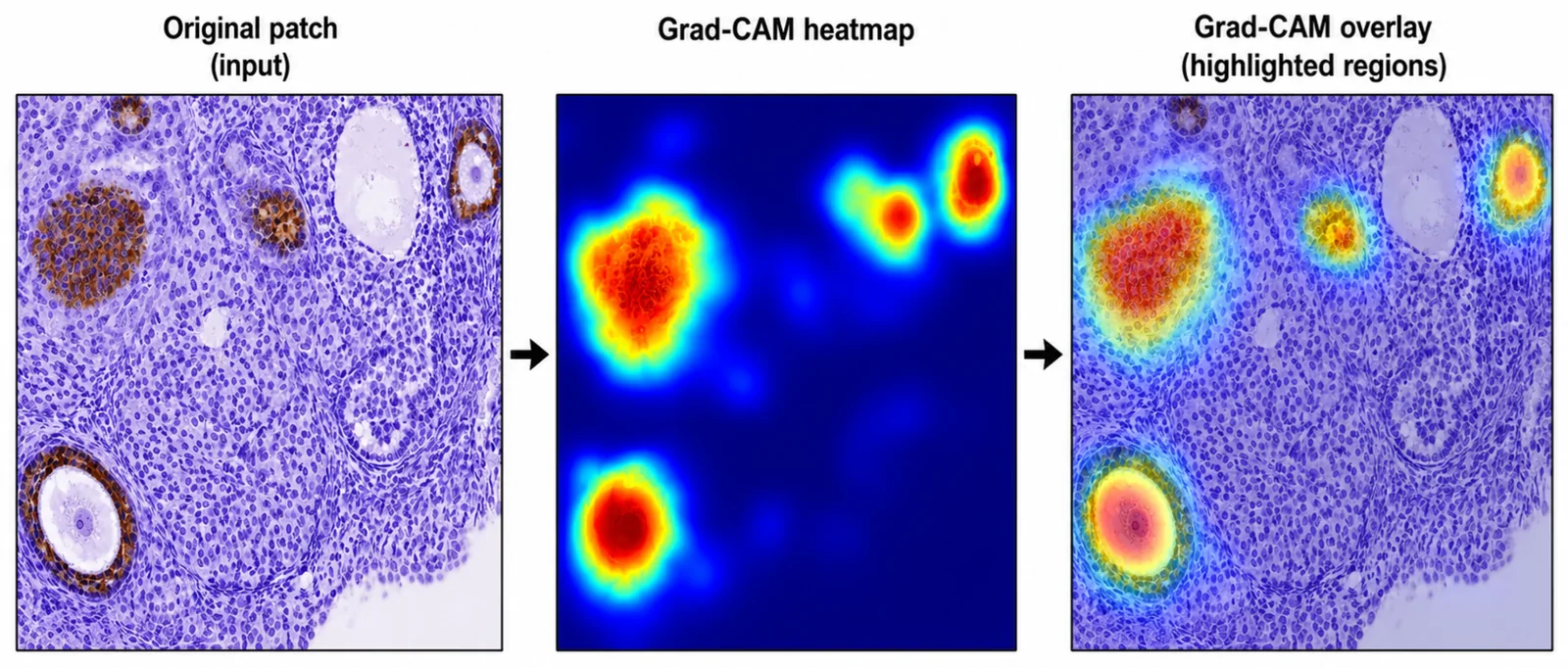
Representative Grad-CAM visualization. The heatmap was resized to the input patch size and overlaid on the original patch image to visualize regions contributing to the model prediction.

**Figure 6:**
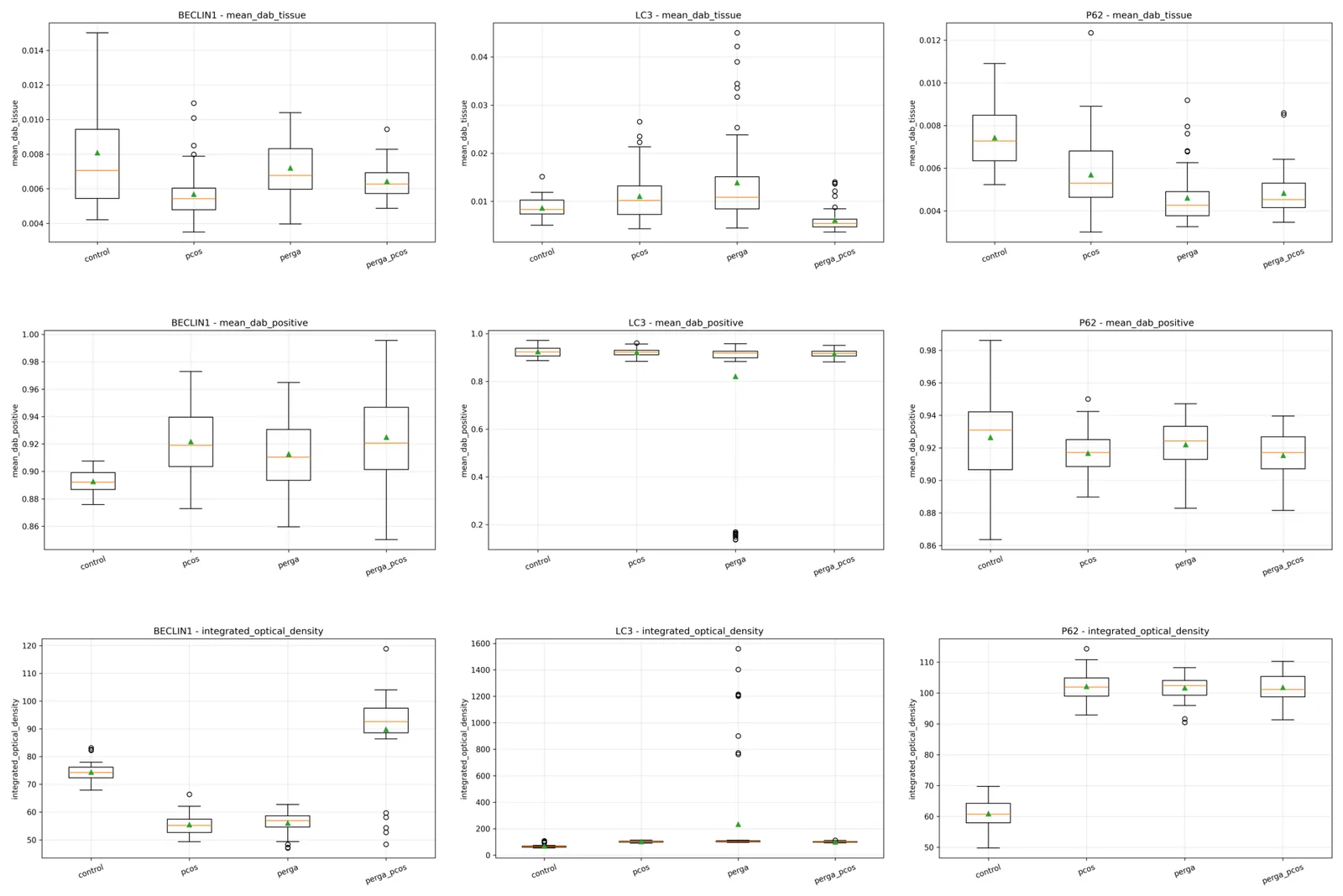
Quantitative DAB analysis of Beclin-1, LC3, and p62 staining across experimental groups. The first row shows tissue-normalized DAB intensity, the second row shows mean DAB intensity in DAB-positive regions, and the third row shows integrated optical density. Green triangles indicate group means, and circular points indicate outliers.

**Figure 7:**
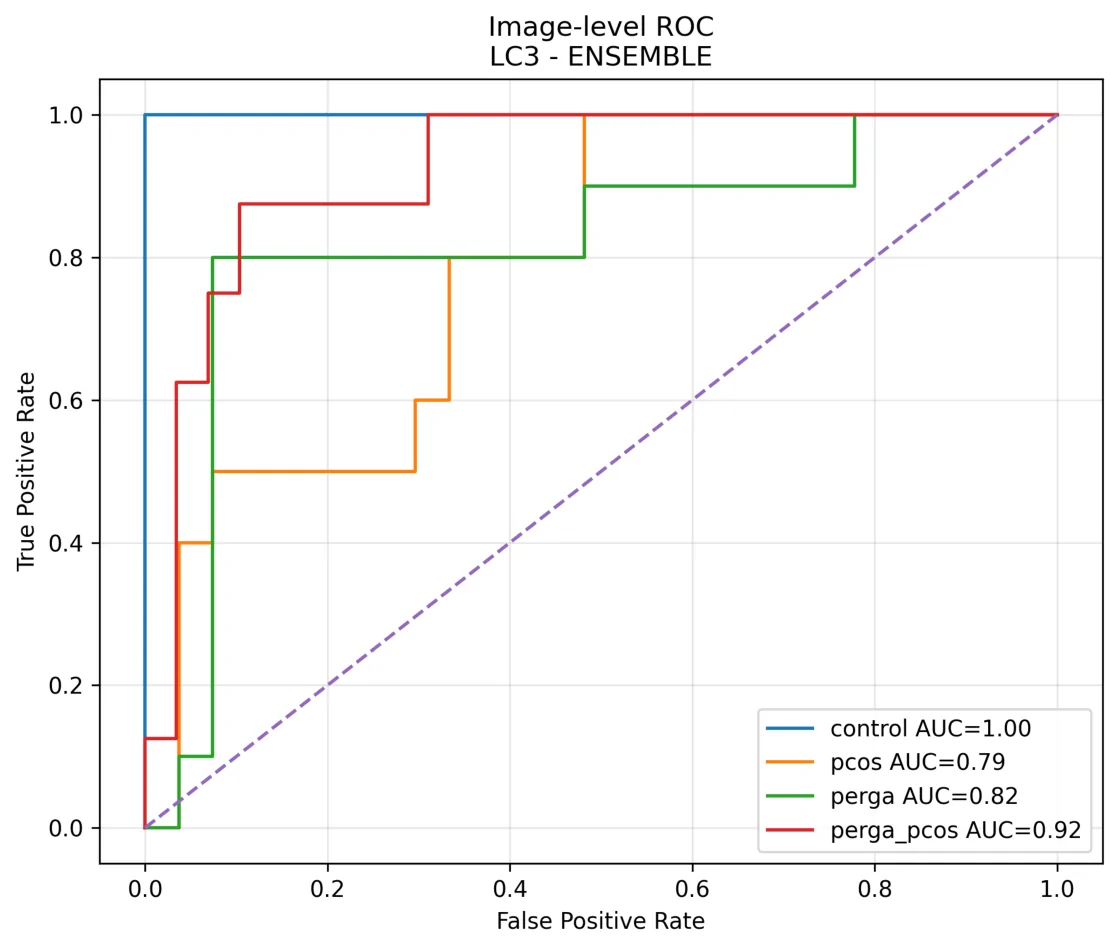
Image-level ROC curves of the ensemble model for the LC3 marker. The dashed diagonal line represents the chance-level reference classifier.

**Figure 8:**
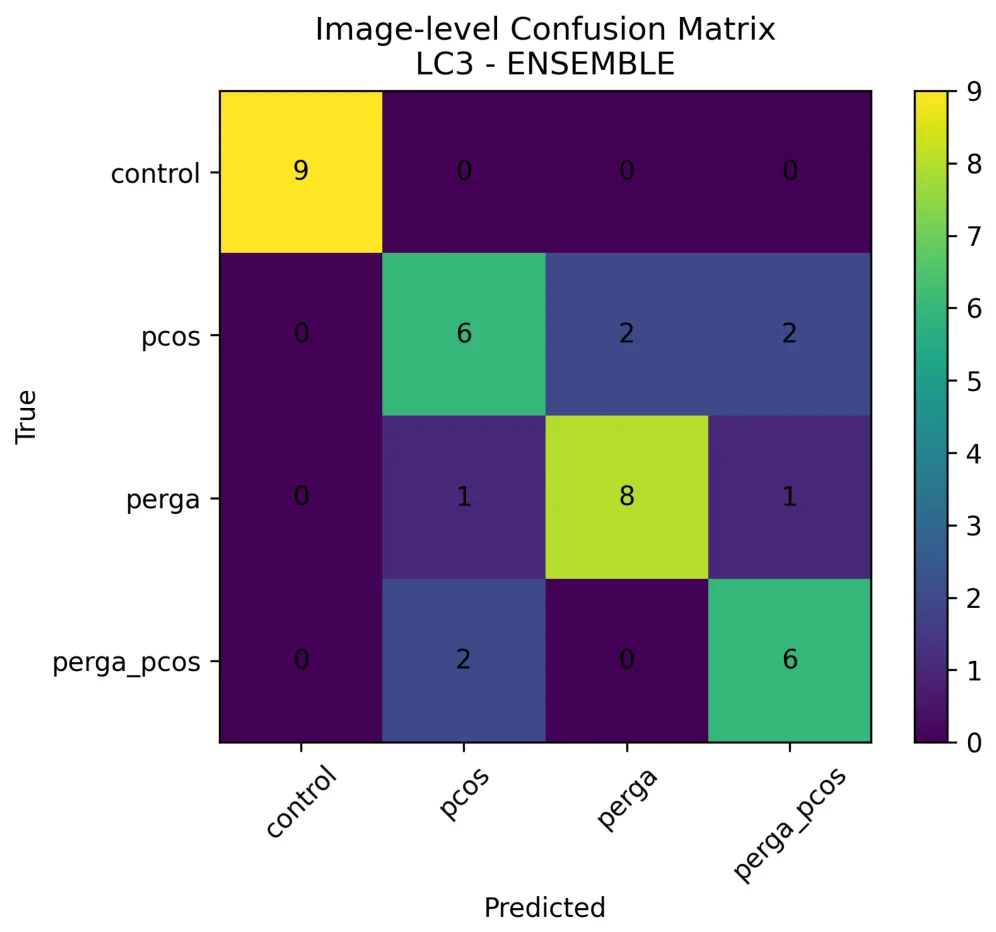
Image-level confusion matrix of the ensemble model for the LC3 marker.

**Figure 9:**
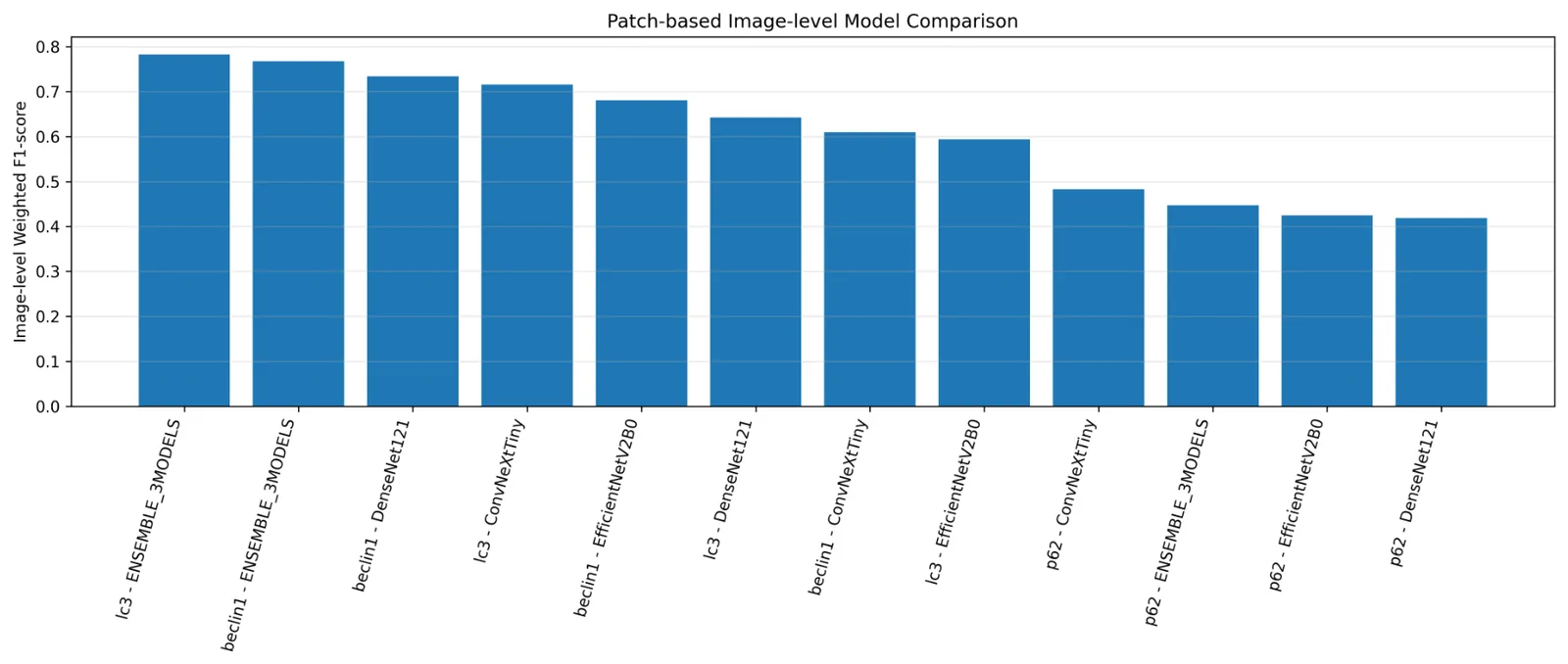
Comparison of F1-scores across different models and markers.

**Figure 10:**
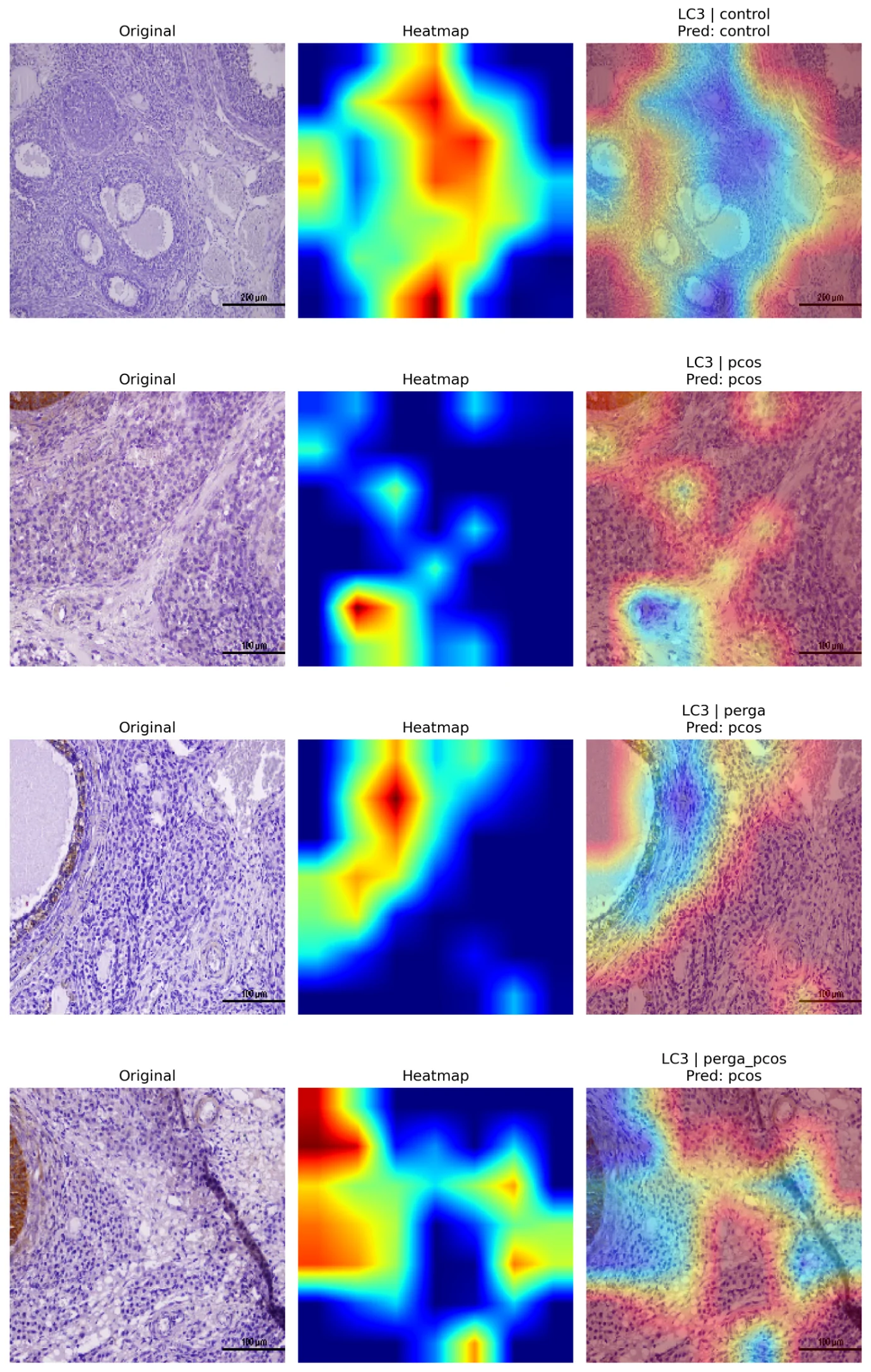
Representative Grad-CAM images of four experimental groups for the LC3 marker. For each group, the original image, Grad-CAM heatmap, and heatmap overlay are shown. Warm colors indicate regions with higher model activation, whereas cooler colors indicate regions with lower activation.

**Table 1:** Dataset Statistics.

Overall Dataset Information				
Total clean IHC images			659	
Total extracted patches			7908	
Patch size			224×224	
**Original Image Distribution**				
**Marker**	**Control**	**PMOS**	**PERGA**	**PERGA + PMOS**
Beclin-1	45	61	49	42
LC3	56	70	63	56
p62	52	65	50	50
**Image Split Statistics**				
**Marker**	**Train**	**Validation**	**Test**	**Total**
Beclin-1	137	30	30	197
LC3	171	37	37	245
p62	151	33	33	217
**Patch Split Statistics**				
**Marker**	**Train**	**Validation**	**Test**	**Total**
Beclin-1	1644	360	360	2364
LC3	2052	444	444	2940
p62	1812	396	396	2604

**Table 2:** Patch-selection criteria used for tissue-rich and DAB-positive patch extraction.

Parameter	Criterion/Value
Patch size	224×224 pixels
Stride	112 pixels
Maximum number of selected patches	Up to 12 patches per image
Tissue mask definition	Grayscale intensity <225
Minimum tissue ratio	0.35
DAB thresholding strategy	75th percentile of DAB-channel values within the tissue mask
Minimum DAB-positive ratio	0.03
Patch quality score	Equation (1)
Fallback criterion	Tissue-rich patches if DAB criterion was not met

**Table 3:** Kruskal-Wallis test results for quantitative DAB measurements.

Marker	Measurement	*p*-Value
p62	positive area percent	p<0.001
p62	mean dab tissue	p<0.001
p62	mean dab positive	p=0.004
p62	integrated optical density	p<0.001
LC3	positive area percent	p<0.001
LC3	mean dab tissue	p<0.001
LC3	mean dab positive	p=0.025
LC3	integrated optical density	p<0.001
Beclin-1	positive area percent	p<0.001
Beclin-1	mean dab tissue	p<0.001
Beclin-1	mean dab positive	p<0.001
Beclin-1	integrated optical density	p<0.001

**Table 4:** Image-level performance comparison of patch-based deep learning models.

Marker	Model	Accuracy	Precision	Recall	F1-Score	AUC
LC3	Ensemble	78.38	78.38	78.38	78.28	88.15
Beclin-1	Ensemble	76.67	77.98	76.67	76.79	92.31
Beclin-1	DenseNet121	73.33	79.89	73.33	73.44	93.63
LC3	ConvNeXtTiny	72.97	73.54	72.97	71.61	90.13
Beclin-1	EfficientNetV2B0	70.00	69.67	70.00	68.10	84.16
LC3	DenseNet121	64.86	65.59	64.86	64.24	86.30
Beclin-1	ConvNeXtTiny	60.00	63.39	60.00	61.03	87.66
LC3	EfficientNetV2B0	62.16	62.48	62.16	59.36	85.90
p62	ConvNeXtTiny	48.48	48.21	48.48	48.28	70.96
p62	Ensemble	45.45	45.49	45.45	44.75	70.79
p62	EfficientNetV2B0	45.45	39.96	45.45	42.45	72.84
p62	DenseNet121	42.42	43.40	42.42	41.84	68.38

## Data Availability

The data supporting the findings of this study are available from the corresponding author upon reasonable request, subject to ethical and institutional restrictions.

## References

[1] Teede H.J., Khomami M.B., Morman R., Laven J.S., Joham A.E., Costello M.F., Patil M., Rees D.A., Berry L., Cree M.G. (2026). Polyendocrine metabolic ovarian syndrome, the new name for polycystic ovary syndrome: A multistep global consensus process. Lancet.

[2] Kobayashi H., Shigetomi H., Matsubara S., Yoshimoto C., Imanaka S. (2024). Role of the mitophagy-apoptosis axis in the pathogenesis of polycystic ovarian syndrome. J. Obstet. Gynaecol. Res..

[3] Zhao Y., Zhao X., Jiang T., Xi H., Jiang Y., Feng X. (2024). A retrospective review on dysregulated autophagy in polycystic ovary syndrome: From pathogenesis to therapeutic strategies. Horm. Metab. Res..

[4] Zhou J., Peng X., Mei S. (2019). Autophagy in ovarian follicular development and atresia. Int. J. Biol. Sci..

[5] Tang X., Gao X., Wu T., Wang S., Gao Y., Zhang J. (2025). Autophagy in ovary: Protective roles, pathological consequences, and unresolved issues. J. Ovarian Res..

[6] Kumariya S., Ubba V., Jha R.K., Gayen J.R. (2021). Autophagy in ovary and polycystic ovary syndrome: Role, dispute and future perspective. Autophagy.

[7] Cheng D., Zheng B., Sheng Y., Zeng Z., Mo Z. (2023). The roles of autophagy in the genesis and development of polycystic ovary syndrome. Reprod. Sci..

[8] Li D., You Y., Bi F., Zhang T., Jiao J., Wang T., Zhou Y., Shen Z., Wang X., Yang Q. (2018). Autophagy is activated in the ovarian tissue of polycystic ovary syndrome. Reproduction.

[9] Li X., Qi J., Zhu Q., He Y., Wang Y., Lu Y., Wu H., Sun Y. (2019). The role of androgen in autophagy of granulosa cells from PCOS. Gynecol. Endocrinol..

[10] Zhang C., Hu J., Wang W., Sun Y., Sun K. (2020). HMGB1-induced aberrant autophagy contributes to insulin resistance in granulosa cells in PCOS. FASEB J..

[11] Tong C., Wu Y., Zhang L., Yu Y. (2022). Insulin resistance, autophagy and apoptosis in patients with polycystic ovary syndrome: Association with PI3K signaling pathway. Front. Endocrinol..

[12] Xu B., Dai W., Liu L., Han H., Zhang J., Du X., Pei X., Fu X. (2022). Metformin ameliorates polycystic ovary syndrome in a rat model by decreasing excessive autophagy in ovarian granulosa cells via the PI3K/AKT/mTOR pathway. Endocr. J..

[13] Ma Y., Ma Y., Li P., Ma F., Yu M., Xu J., Yang Y. (2025). Wnt5a alleviates the symptoms of PCOS by modulating PI3K/AKT/mTOR pathway-mediated autophagy in granulosa cells. Cell. Signal..

[14] Jin R., Chen A., Ye Y., Ren Y., Lu J., Xuan F., Zhou W. (2024). Effect of berberine combined with metformin on autophagy in polycystic ovary syndrome by regulating AMPK/AKT/mTOR pathway. Mol. Reprod. Dev..

[15] Rizzardi A.E., Johnson A.T., Vogel R.I., Pambuccian S.E., Henriksen J., Skubitz A.P., Metzger G.J., Schmechel S.C. (2012). Quantitative comparison of immunohistochemical staining measured by digital image analysis versus pathologist visual scoring. Diagn. Pathol..

[16] Varghese F., Bukhari A.B., Malhotra R., De A. (2014). IHC Profiler: An open source plugin for the quantitative evaluation and automated scoring of immunohistochemistry images of human tissue samples. PLoS ONE.

[17] Khan A.M., Rajpoot N., Treanor D., Magee D. (2014). A nonlinear mapping approach to stain normalization in digital histopathology images using image-specific color deconvolution. IEEE Trans. Biomed. Eng..

[18] Banerji S., Mitra S. (2022). Deep learning in histopathology: A review. Wiley Interdiscip. Rev. Data Min. Knowl. Discov..

[19] Komura D., Ochi M., Ishikawa S. (2025). Machine learning methods for histopathological image analysis: Updates in 2024. Comput. Struct. Biotechnol. J..

[20] Gadermayr M., Tschuchnig M. (2024). Multiple instance learning for digital pathology: A review of the state-of-the-art, limitations & future potential. Comput. Med. Imaging Graph..

[21] Köseoğlu E. (2024). Deneysel Polikistik over Sendromu Modelinde Perga’nın Olası Koruyucu Etkisi. Master’s Thesis.

[22] Jalouli M., Harrath A.H., Al-Zharani M., Rahman M.A. (2026). Autophagy Modulation in Cancer Immunotherapy, Emerging Molecular Targets and Drug Selection Strategies. Int. J. Mol. Sci..

[23] Saha P., Kumar S., Datta K., Tyagi R.K. (2021). Upsurge in autophagy, associated with mifepristone-treated polycystic ovarian condition, is reversed upon thymoquinone treatment. J. Steroid Biochem. Mol. Biol..

[24] Huang G., Liu Z., Van Der Maaten L., Weinberger K.Q. (2017). Densely connected convolutional networks. Proceedings of the 2017 IEEE Conference on Computer Vision and Pattern Recognition (CVPR), Honolulu, HI, USA, 21–26 July 2017.

[25] Tan M., Le Q. (2021). EfficientNetV2: Smaller models and faster training. Proceedings of the 38th International Conference on Machine Learning, Virtual, 18–24 July 2021.

[26] Liu Z., Mao H., Wu C., Feichtenhofer C., Darrell T., Xie S. (2022). A convnet for the 2020s. Proceedings of the 2022 IEEE/CVF Conference on Computer Vision and Pattern Recognition (CVPR).

